# Metal complexes of benzimidazole-derived as potential anti-cancer agents: synthesis, characterization, combined experimental and computational studies

**DOI:** 10.1098/rsos.220659

**Published:** 2022-09-21

**Authors:** Van-Thanh Nguyen, Thi-Kim-Chi Huynh, Gia-Thien-Thanh Ho, Thi-Hong-An Nguyen, Thi Le Anh Nguyen, Duy Quang Dao, Tam V. T. Mai, Lam K. Huynh, Thi-Kim-Dung Hoang

**Affiliations:** ^1^ Institute of Chemical Technology – VAST, 1A Thanh Loc 29 Street, Thanh Loc Ward, District 12, Ho Chi Minh City 700000, Vietnam; ^2^ Graduate University of Science and Technology – VAST, 18 Hoang Quoc Viet Street, Nghia Do Ward, Cau Giay District, Hanoi 100000, Vietnam; ^3^ Ton Duc Thang University, 19 Nguyen Huu Tho Street, Tan Phong Ward, District 7, Ho Chi Minh City 700000, Vietnam; ^4^ Institute of Research and Development, Duy Tan University, Da Nang 50000, Vietnam; ^5^ Institute for Computational Science and Technology, SBI Building, Quang Trung Software City, Tan Chanh Hiep Ward, District 12, Ho Chi Minh City 700000, Vietnam; ^6^ University of Science, Ho Chi Minh City, 227 Nguyen Van Cu Street, Ward 4, District 5, Ho Chi Minh City 700000, Vietnam; ^7^ Vietnam National University, Ho Chi Minh City 700000, Vietnam; ^8^ International University, Block 6, Linh Trung Ward, Thu Duc District, Ho Chi Minh City 700000, Vietnam

**Keywords:** benzimidazole, metal complex, characterization, experiment, calculation, anti-cancer agent

## Abstract

In this study, a series of 14 Cu (II), Zn (II), Ni (II) and Ag (I) complexes containing bis-benzimidazole derivatives were successfully designed and synthesized from 2-(1*H*-benzimidazole-2-yl)-phenol derivatives and corresponding metal salt solutions. The compound structures were identified by FT-IR, ^1^H-NMR, powder X-ray diffraction and ESI-MS analyses, and the presence of the metal in the complexes was confirmed by ultraviolet-visible spectroscopy and ICP optical emission spectrometry. Electronic structure calculations were also carried out to describe the detailed structures in addition to the electronic absorption spectra of the ligands. The cytotoxic activity of the complexes was evaluated against three human cancer cell lines: lung (A549), breast (MDA-MB-231) and prostate (PC3) cancer cells. All complexes inhibited anti-proliferative cancer cells better than free ligands, especially Zn (II) and Ag (I) complexes, which are most sensitive to MDA-MB-231 cells. In addition, showing the growth inhibition of three cancer cell lines with IC_50_ < 10.4 µM, complexes **C_1_**, **C_3_** and **C_14_** could be considered potential multi-targeted anti-cancer agents.

## 1. Introduction

For many decades, cancer has been a major disease worldwide and threatens human lives; thus, anti-cancer drugs have received much attention from scientists. Since the serendipitous discovery of cisplatin shows anti-proliferative activity, metallodrugs have prompted an exciting research topic within medicinal chemistry as promising agents for cancer treatment [1]. Cisplatin and its analogues, such as carboplatin and oxaliplatin, have been widely used to treat various solid tumours, including mesothelioma, neuroblastoma [2], ovarian cancer [3], lung cancer [4], brain tumour, head and neck cancer and testicular cancer [5]. Despite therapeutic success in clinical applications, the main disadvantages of platinum-based drugs are undesirable side effects (e.g. nephrotoxicity, hepatotoxicity, ototoxicity and neurotoxicity) and drug resistance [6]. Therefore, there has been much effort to search for non-platinum metal compounds to minimize their side effects and drug resistance, as well as to retain high activity.

In an attempt to replace platinum-based drugs, the transition metals, particularly copper, zinc and nickel, are the most investigated classes due to their biocompatibility and endogenous presence in the living system as cofactors in numerous enzymes [7]. Moreover, transition metal-based therapeutic agents tend to mimic phytochemicals with low toxicity and effectively bind to DNA, which leads to its cleavage as one of the characteristics of effective anti-cancer drugs such as cisplatin [8,9]. Likewise, depending on their coordination numbers, metal ions are likely to create various geometries, including linear, trigonal planar, tetrahedral and octahedral structures. Structural diversity has been shown to have an enormous effect on the design of novel drugs that enhance the biological activity for cancer treatment [10].

Transition metal-based complexes such as copper (II), zinc (II), cobalt (II), ruthenium (II), nickel (II), silver (I) and gold (I) ions have attracted significant interest as promising anti-tumour therapeutic agents [11]. The copper (II) complex with 2-[(1*H*-benzimidazol-2-ylimino)-methyl]-6-methoxyphenol ligand exhibits a profound potential against MCF-7 human breast cancer cells using the MTT method and simultaneously releases chronic pain in cancer patients in the last stage as non-steroidal inflammatory drugs [12]. Similarly, Au (I)-bis-N-acyclic carbene reveals inhibition of cell growth both for FLG 29.1 (human acute myeloid leukaemia) and HCT-116 (human colorectal adenocarcinoma), with the IC_50_ values 2.59 and 1.81 µM, respectively, exceeding the reference drug cisplatin, with IC_50_ the values 24.41 and 25.52 µM, respectively [13]. On the other hand, metal-organic hybridization is often more effective than free organic compounds. For example, the kaempferol-Zn (II) complex shows two times higher anti-neoplastic activity against EC9706 cells than kaempferol [14].

Although metal ions play a crucial role in synergistic cell-killing effects [15], organic frameworks significantly contribute to the cytotoxic activity, especially aromatic Schiff bases [16]. These bases are likely to easily coordinate with a variety of metal ions via imine groups on heterocyclic compounds and maintain their stability in many oxidation states [17,18]. In this context**,** benzimidazole and its derivatives emerge as a promising candidate to coordinate with metal ions due to the conjugated pi-system, planarity, mimicry of imidazole function in proteins, and the presence in the body as a part of vitamin B12 [19,20]. The medicinal significance of this ligand can be easily observed by its being a commercial drug, for example, Abemaciclib—an anti-tumour agent [21]. Moreover, these derivatives show many pharmacological activities, including anti-microbial, anti-ulcerative [22], anti-hypertensive [23], anti-viral [24], anti-inflammatory, anti-protozoal and anti-cancer activities [25]. Thus, the presence of benzimidazole-derived ligands in the structural design of the complexes may biologically consolidate the anti-proliferative activities.

For these purposes, we designed and synthesized a series of novel Cu (II), Zn (II), Ni (II) and Ag (I) complexes with benzimidazole derivatives. All ligands and complexes were characterized using different spectroscopic and spectrometric analyses. Furthermore, the cytotoxic activities of free ligands and complexes were evaluated for their *in vitro* anti-cancer activity in human cancer cell lines, such as lung (A549), breast (MDA-MB-231) and prostate (PC3). In our study, although, many compounds have been prepared in several reports for the last few years [26–30], this work has been the first report of metal complexes of 2-(1*H*-benzimidazol-2-yl)-phenoxy-derived scaffolds with cytotoxic activity in human cancer cell lines.

## 2. Materials and methods

### 2.1. General experimental procedures

All reagents used in the reaction were purchased from Merck (Germany) and Acros (Belgium). Cu(CH_3_COO)_2_.H_2_O, Zn(CH_3_COO)_2_.2H_2_O, Ni(CH_3_COO)_2_.4H_2_O and AgNO_3_ solutions were provided by Johnson Matthey Chemicals Inc. The solvents used in this study were provided by Xilong (China) and Chemsol (Vietnam) and used without further purification. Melting points were performed on an instrument of the Electrothermal IA 9000 series and were uncorrected. FT-IR spectroscopy was performed using a Bruker Tensor 27 instrument, and the absorption bands were determined in wavenumber (cm^−1^). UV-Vis absorption spectra were obtained using UV-Vis Jasco V-630 in DMSO or acetonitrile (MeCN) solutions. The metal contents were determined using an ICP-OES system (PerkinElmer Optima 2100 DV). ^1^H-NMR and ^13^C-NMR spectra were recorded in (CD_3_)_2_SO on a Bruker AM0 FT-NMR spectrometer at 500 MHz (^1^H-NMR) and 125 MHz (^13^C-NMR). The chemical shifts were expressed in *δ* (ppm) relative to tetramethylsilane as an internal standard. The following data were obtained: chemical shift, integration, multiplicity (s = singlet, d = doublet, t = triplet, q = quartet, m = multiplet, dd = doublet of doublet), and coupling constants (*J*, Hz) in Hz and position. ESI-HRMS was analysed on a Sciex X500R system with quadrupole time-of-flight mass spectrometer in electrospray ionization (ESI) mode. Powder X-ray diffraction (PXRD) was carried out on an XRD EQUINOX 5000 diffractometer (Thermo Scientific) with Cu-K*_α_* radiation at a wavelength of 0.154 nm. The diffraction angles (2*θ*) were scanned from 5° to 120° (excluding C_1_, from 5° to 80°) with a step size of 0.015° and a time per step of 1 s.

### 2.2. Synthesis of ligands L_1_–L_5_

The synthesis of ligands **L_1_**–**L_5_** was described in our previous reports [31,32], and for convenience of information searching, their identified characteristics are listed below.

#### 2.2.1. 2-(1H-benzimidazol-2-yl)-phenol (**L_1_**)

The product was a light-yellow powder. Yield: 92%; M.p.: 230–231°C; IR (KBr, cm^−1^): *ν* (-NH) & *ν* (-OH) = 3324, *ν* (-C = N) 1631, *ν* (C = C) 1589, *δ* (-NH) 1491, *ν* (C-O) 1261; ESI-HRMS (DMSO, *m/z*): calcd. for C_13_H_10_N_2_O: [M + H]^+^ = 211.0793, found 211.0877; UV-Vis (DMSO, nm) (*ε*, cm^−1^ mol^−1^ dm^3^): 293 (9300), 319 (13 300), 334 (12 100); ^1^H-NMR (500 MHz, DMSO-*d_6_*, *δ*): 13.17 (s, 1H, -OH), 13.14 (s, 1H, -NH), 8.06–8.04 (dd, 1H, Ar-H, *J* = 1.5 Hz, *J* = 7.5 Hz), 7.72–7.71 (d, 1H, Ar-H, *J* = 6 Hz), 7.61–7.59 (d, 1H, Ar-H, *J* = 6.5 Hz), 7.40–7.37 (m, 1H, Ar-H), 7.29 (s, 2H, Ar-H), 7.05–7.00 (m, 2H, Ar-H). ^13^C-NMR (125 MHz, DMSO-d6, *δ* ppm): 157.85, 151.63, 140.80, 133.10, 131.66, 126.14, 123.21, 122.34, 119.04, 117.89, 117.11, 112.53, 111.45.

#### 2.2.2. 2-(5-methyl-benzimidazol-2-yl)-phenol (**L_2_**)

The product was a light brown powder. Yield: 90%; M.p.: 248–249°C; IR (KBr, cm^−1^): *ν* (-NH) & *ν* (-OH) 3237, *ν* (-C = N) 1639, *ν* (C = C) 1540, *δ* (-NH) 1487, *ν* (C-O) 1261; ESI-HRMS: calcd. for C_14_H_12_N_2_O: [M + H]^+^ = 225.1027, found [M + H]^+^ = 225.1039; UV-Vis (DMSO, nm) (*ε*, cm^−1^ mol^−1^ dm^3^): 296 (9800), 322 (15 200), 337 (14 100); ^1^H-NMR (500 MHz, DMSO-*d_6_*, *δ*): 13.18 (s, 1H, -OH), 13.05 (s, 1H, −NH), 8.04–8.02 (dd, 1H, Ar-H, *J* = 1.5 Hz, *J* = 7.5 Hz), 7.59 (s, 1H, Ar-H), 7.511 (s, 1H, Ar-H), 7.39–7.36 (m, 1H, Ar-H), 7.12 (s, 1H, Ar-H), 7.04–6.99 (m, 1H, Ar-H), 2.46 (s, 3H, -CH_3_). ^13^C-NMR (125 MHz, DMSO-d_6_, *δ* ppm): 157.89, 131.47, 126.11, 125.96, 123.90, 118.98, 117.64, 117.07, 112.64, 111.12, 21.20.

#### 2.2.3. 2-(5-chloro-benzimidazol-2-yl)-phenol (**L_3_**)

The product was a light-yellow powder. Yield: 68%; M.p.: 240–241°C. IR (KBr, cm^−1^): *ν* (-N-H) & *ν* (-OH) 3330, *ν* (-C = N) 1633, *ν* (C = C) 1585, *δ* (-NH) 1489, *ν* (C-O) 1257; ESI-HRMS: calcd. for C_13_H_9_N_2_OCl: [M + H]^+^ = 245.0481, found 245.0463; UV-Vis (DMSO, nm) (*ε*, cm^−1^ mol^−1^ dm^3^): 297 (9400), 322 (16 300), 336 (15 000); ^1^H-NMR (500 MHz, DMSO-*d_6_, δ*): 12.73 (s, 1H, -NH/-OH), 8.06–8.04 (d, 1H, Ar-H, *J* = 1 Hz), 7.66 (s, 1H, Ar-H), 7.41–738 (m, 1H, Ar-H), 7.30–7.28 (t, 1H, Ar-H, *J* = 7 Hz), 7.06–7.01 (m, 2H, Ar-H). ^13^C-NMR (125 MHz, DMSO-d6, *δ* ppm): 157.79, 152.80, 132.04, 126.56, 122.99, 119.26, 117.19, 112.51.

#### 2.2.4. 2-(2-hydroxy-phenyl)-1H-benzimidazol-5-yl]-phenyl-methanone (**L_4_**)

The product was a light-yellow powder. Yield: 75% M.p.: 262–263°C; IR (KBr, cm^−1^): *ν* (-N-H) & *ν* (-OH) 3296, *ν* (-C = N) 1613, *ν* (C = C) 1573, *δ* (-NH) 1487, *ν* (C-O) 1259; ESI- HRMS: Exact mass of molecular ion *m/z* [M + H]^+^ = 315.1131, calcd. for C_20_H_14_N_2_O_2_: [M + H]^+^ = 315.1055; UV-Vis (DMSO, nm) (*ε*, cm^−1^ mol^−1^ dm^3^): 337 (16 200); ^1^H-NMR (500 MHz, DMSO-*d_6_, δ*): 13.38 (s, 1H, -OH), 12.68 (s, 1H, -NH), 8.09–8.08 (d, 1H, Ar-H, *J* = 8.5 Hz), 8.02 (s, 1H, Ar-H), 7.83–7.75 (m, 4H, Ar-H), 7.70–7.67 (t, 1H, Ar-H, *J* = 7.5 Hz), 7.60–7.57 (t, 2H, Ar-H, *J* = 7.5 Hz), 7.44–7.40 (m, 1H, Ar-H), 7.08–7.03 (m, 2H, Ar-H, *J* = 8 Hz). ^13^C-NMR (125 MHz, DMSO-d6, *δ* ppm): 195.45, 157.89, 137.93, 132.32, 132.12, 131.53, 129.41, 128.42, 126.80, 119.35, 117.25, 112.51.

#### 2.2.5. 5-chloro-2-(2-trifluromethyl-phenyl)-1H-benzimidazole (**L_5_**)

The product was a light-grey powder. Yield: 83%. M.p.: 173–174°C. IR (KBr, cm^−1^): *ν* (-N-H) 3052, *ν* (-C = N) 1620, *ν* (C = C) 1583, *δ* (-NH) 1433; ESI-HRMS: calcd. for C_20_H_14_N_2_O_2_: [M + H]^+^ = 297.0406, found [M + H]^+^ = 297.0408; UV-Vis (MeCN, nm) (*ε*, cm^−1^ mol^−1^ dm^3^): 206 (24 000), 292 (11 800); ^1^H-NMR (500 MHz, DMSO-*d_6_, δ)*: 12.99 (s, 1H, -NH), 7.95 (d, 1H, Ar-H, *J* = 7.5 Hz), 7.76–7.86 (m, 3H, Ar-H), 7.69 (s, 1H, Ar-H), 7.63 (s, 1H, Ar-H, *J* = 6.5 Hz), 7.26–7.28 (dd, 1H, Ar-H, *J* = 8.5, *J* = 2 Hz). ^3^C-NMR (125 MHz, DMSO-d6, *δ* ppm): 150.80, 132.44, 132.17, 130.46, 129.68, 129.67, 128.24, 127.75, 127.51, 126.94, 126.70, 126.66, 126.62, 126.58, 124.76, 122.58, 120.41.

### 2.3. Synthesis of complexes C_1_–C_14_

#### 2.3.1. 2-(1H-benzimidazol-2-yl)-phenol zinc (II) (**C_1_**)

Ligand **L_1_** (0.252 g, 1.2 mmol) and Zn(CH_3_COO)_2_.4H_2_O (0.1314 g, 0.6 mmol) were completely dissolved in ethanol (15 ml) and stirred vigorously at room temperature. The colour of the solution changed, and the precipitate appeared immediately. The reaction was monitored by TLC. After a 24 h reaction, the precipitate was filtered and washed with water and cool ethanol, and the product was crystallized in DMF. The product was a white powder. Yield: 91%; M.p.: greater than 300°C; IR (KBr, cm^−1^): *ν* (-NH) 3108, *ν* (-C = N) 1625, *ν* (C = C) 1531, *δ* (-NH) 1477, *ν* (C-O) 1252, *ν* (Zn-O) 539, *ν* (Zn-N) 427; ESI-HRMS: calcd. for C_26_H_18_N_4_O_2_Zn: [M + H]^+^ = 483.0799, found [M + H]^+^ = 483.0718; UV-Vis (MeCN, nm) (*ε*, cm^−1^ mol^−1^ dm^3^): 212 (33 500), 291 (7100), 317 (12 900), 330 (15 000), 363 (1500); ^1^H-NMR (500 MHz, DMSO-*d_6_, δ*): 8.00–7.98 (d, 1H, Ar-H, *J* = 7.5 Hz), 7.59–7.57 (d, 1H, Ar-H, *J* = 8 Hz), 7.29–7.22 (m, 2H, Ar-H), 7.08–7.05 (t, 2H, Ar-H, *J* = 8 Hz), 6.82–6.80 (d, 1H, Ar-H, *J* = 8 Hz), 6.70–6.67 (t, 1H, Ar-H, *J* = 7 Hz). ICP: calcd. for Zn = 13.4%, found Zn = 13.5%.

Similar to the procedure above, complexes **C_2_**–**C_4_** were synthesized from ligands **L_2_**–**L_4_** and Zn(CH_3_COO)_2_.4H_2_O.

#### 2.3.2. 2-(5-methyl-benzimidazol-2-yl)-phenol zinc (II) (**C_2_**)

White powder, yield: 92%; M.p.: greater than 300°C; IR (KBr, cm^−1^): *ν* (-NH) 3054, *ν* (-C = N) 1604, *ν* (C = C) 1532, *δ* (-NH) 1477, *ν* (C-O) 1311, *ν* (Zn-O) 517, *ν* (Zn-N) 428; ESI- HRMS: calcd. for C_28_H_22_N_4_O_2_Zn: [M + H]^+^ = 511.1112, found [M + H]^+^ = 511.0798; UV-Vis (MeCN, nm) (*ε*, cm^−1^ mol^−1^ dm^3^): 241 (47 700), 290 (7100), 318 (7000), 334 (7800), 365 (3200); ^1^H-NMR (500 MHz, DMSO-*d_6_, δ*): 7.97 (s, 1H, Ar-H), 7.48–7.26 (m, 3H, Ar-H), 7.09–7.06 (d, 1H, Ar-H), 6.88–6.68 (m, 3H, Ar-H), 2.15–2.53 (3H, -CH_3_).

#### 2.3.3. 2-(5-chloro-benzimidazol-2-yl)-phenol zinc (II) (**C_3_**)

White powder, yield: 61%; M.p.: greater than 300°C; IR (KBr, cm^−1^): *ν* (-NH) 3058, *ν* (-C = N) 1603, *ν* (C = C) 1526, *δ* (-NH) 1477, *ν* (C-O) 1249, *ν* (Zn-O) 508, *ν* (Zn-N) 422; ESI- HRMS: calcd. for C_26_H_16_N_4_O_2_Cl_2_Zn: [M + H]^+^ = 551.0020, found [M + H]^+^ = 551.0064; UV-Vis (MeCN, nm) (*ε*, cm^−1^ mol^−1^ dm^3^): 242 (42 000), 319 (8100), 335 (7800); ^1^H-NMR (500 MHz, DMSO-*d_6_, δ*): 13.39 (s, 1H, -NH), 7.95 (s, 1H, Ar-H), 7.56–7.54 (d, 1H, Ar-H), 7.24 (s, 2H, Ar-H), 7.10 (s, 1H, Ar-H) 6.77(s, 1H, Ar-H), 6.63 (s, 1H, Ar-H).

#### 2.3.4. [2-(2-hydroxy-phenyl)-1H-benzimidazol-5-yl]-phenyl-methanone zinc (II) (**C_4_**)

White powder, yield: 78%; M.p.: greater than 300°C; IR (KBr, cm^−1^): *ν* (-NH) 3087, *ν* (-C = N) 1622, *ν* (C = C) 1526, *δ* (-NH) 1477, *ν* (C-O) 1247, *ν* (Zn-O) 503, *ν* (Zn-N) 435; ESI-HRMS: calcd. for C_40_H_26_N_4_NaO_4_Zn: [M + Na]^+^ = 713.1143, found [M + Na]^+^ = 713.1153; UV-Vis (MeCN, nm) (*ε*, cm^−1^ mol^−1^ dm^3^): 237 (42 800), 375 (28 800); ^1^H-NMR (500 MHz, DMSO-*d_6_, δ*): 7.90 (s, 2H,Ar-H), 7.71–7.38 (m, 8H, Ar-H), 7.21 (s, 1H, Ar-H), 6.70–6.61(m, 2H, Ar-H). ICP: calcd. for Zn = 9.40%, found Zn = 9.01%.

#### 2.3.5. 2-(1H-benzimidazol-2-yl)-phenol copper (II) (**C_5_**)

A mixture of ligand **L_1_** (0.252 g, 1.2 mmol) and Cu(CH_3_COO)_2_.H_2_O (0.12 g, 0.6 mmol) in distilled ethanol (15 ml) was stirred at room temperature. The colour of the solution changed and the precipitate appeared immediately. The reaction was monitored by TLC. After a 24 h reaction, the precipitate was collected by filtration and crystallized from DMF. The given product is a brown powder, yield: 89%; M.p.: greater than 300°C; IR (KBr, cm^−1^): *ν* (-NH) 3057, *ν* (-C = N) 1602, *ν* (C = C) 1554, *δ* (-NH) 1478, *ν* (C-O) 1259, *ν* (Cu-O) 474, *ν* (Cu-N) 438; ESI-HRMS: calcd. for C_26_H_18_N_4_O_2_Cu: [M + H]^+^ = 482.0804, found [M + H]^+^ = 482.0751; UV-Vis (DMSO, nm) (*ε*, cm^−1^ mol^−1^ dm^3^) (4.15×10^−^^5^M): 297 (17 100), 318 (12 100), 334 (13 500), 356 (21 800), 368 (21 100), 640 (130); ICP: calcd. for Cu = 13.27%, found Cu = 12.82%.

Similar to the procedure above, compounds **C_6_**–**C_8_** were synthesized from ligands **L_2_**–**L_4_** and Cu(CH_3_COO)_2_.H_2_O.

#### 2.3.6. 2-(5-methyl-benzimidazol-2-yl)-phenol copper (II) (**C_6_**)

Brown powder, yield: 62%; M.p.: greater than 300°C; IR (KBr, cm^−1^): *ν* (-NH) 3049, *ν* (-C = N) 1604, *ν* (C = C) 1535, *δ* (-NH) 1479, *ν* (C-O) 1261, *ν* (Cu-O) 469, *ν* (Cu-N) 438; ESI-HRMS: calcd. for C_28_H_22_N_4_O_2_Cu: [M + H]^+^ = 510.1117, found [M + H]^+^ = 510.1112; UV-Vis (DMSO, nm) (*ε*, cm^−1^ mol^−1^ dm^3^): 300 (18 900), 323 (13 200), 358 (14 900), 371 (14 200), 641(90); ICP: calcd. for Cu = 12.55%, found Cu = 12.99%.

#### 2.3.7. 2-(5-chloro-benzimidazol-2-yl)-phenol copper (II) (**C_7_**)

Brown powder, yield: 96%; M.p.: greater than 300°C; IR (KBr, cm^−1^): *ν* (-NH) 3113, *ν* (-C = N) 1602, *ν* (C = C) 1554, *δ* (-NH) 1476, *ν* (C-O) 1255, *ν* (Cu-O) 523, *ν* (Cu-N) 439; ESI-HRMS: calcd. for C_26_H_16_N_4_O_2_Cl_2_Cu: [M + H]^+^ = 551.0049, found [M + H]^+^ = 551.0226; UV-Vis (DMSO, nm) (*ε*, cm^−1^ mol^−1^ dm^3^) (3.63×10^−^^5^M): 302 (15 100), 321 (10 900), 337 (19 200), 359 (24 600), 631(130); ICP: calcd. for Cu = 11.60%, found Cu = 8.90%.

#### 2.3.8. [2-(2-hydroxy-phenyl)-1H-benzimidazol-5-yl]-phenyl-methanone copper (II) (**C_8_**)

Brown powder, yield: 78%; M.p.: greater than 300°C; IR (KBr, cm^−1^): *ν* (-NH) 3052, *ν* (-C = N) 1617, *ν* (C = C) 1550, *δ* (-NH) 1475, *ν* (C-O) 1254, *ν* (Cu-O) 476, *ν* (Cu-N) 441; ESI-HRMS: calcd. For C_40_H_26_N_4_NaO_4_Zn: [M + Na]^+^ = 712.1147, found [M + Na]^+^ = 712.1145; UV-Vis (DMSO, nm) (*ε*, cm^−1^ mol^−1^ dm^3^): 380 (25 000), 656 (80); ICP: calcd. for Cu = 9.28%, found Cu = 8.10%.

#### 2.3.9. 2-(1H-benzimidazol-2-yl)-phenol nickel (II) (**C_9_**)

Ligand **L_1_** (0.252 g, 1.2 mmol) was dissolved in distilled ethanol (10 ml) in a two-neck round-bottom flask. Then, a solution of Ni(CH_3_COO)_2_.4H_2_O (0.1488 g, 0.6 mmol) was added, and the colour of the mixture changed immediately. The mixture was stirred at room temperature. After 3 days, the precipitate was filtered and crystallized from DMF. The given product is a yellow powder, yield: 68%; M.p.: greater than 300°C; IR (KBr, cm^−1^): *ν* (-NH) 3098, *ν* (-C = N) 1605, *ν* (C = C) 1564, *δ* (-NH) 1481, *ν* (C-O) 1262, *ν* (Ni-O) 468, *ν* (Ni-N) 441; ESI-HRMS: calcd. for C_26_H_18_N_4_O_2_Ni: [M + Na]^+^ = 499.0680, found [M + Na]^+^ = 499.0521; UV-Vis (DMSO, nm) (*ε*, cm^−1^ mol^−1^ dm^3^): 292 (16 100), 318 (15 300), 332 (15 600), 370 (15 600), 589 (70), 695 (50); ICP calcd. for Ni = 12.18%, found Ni = 11.60%.

Similar to the procedure above, compounds **C_10_**–**C_12_** were synthesized from the ligands **L_2_**–**L_4_** and Ni(CH_3_COO)_2_.4H_2_O.

#### 2.3.10. 2-(5-methyl-benzimidazol-2-yl)-phenol nickel (II) (**C_10_**)

Yellow powder, yield: 71%; M.p.: greater than 300°C; IR (KBr, cm^−1^): *ν* (-NH) 3056, *ν* (-C = N) 1605, *ν* (C = C) 1539, *δ* (-NH) 1481, *ν* (C-O) 1262, *ν* (Ni-O) 469, *ν* (Ni-N) 441; ESI-HRMS: calcd. for C_28_H_22_N_4_O_2_Ni: [M-H]^−^ = 503.1017, found [M-H]^−^ = 503.0702; UV-Vis (DMSO, nm) (*ε*, cm^−1^ mol^−1^ dm^3^): 295 (12 400), 303 (12 800), 321 (12 500), 338 (16 900), 370 (17 800), 586 (30), 694 (40)

#### 2.3.11. 2-(5-chloro-benzimidazol-2-yl)-phenol nickel (II) (**C_11_**)

Yellow powder, yield: 65%; M.p.: greater than 300°C; IR (KBr, cm^−1^): *ν* (- NH) 3041, *ν* (-C = N) 1605, *ν* (C = C) 1563, *δ* (-NH) 1479, *ν* (C-O) 1253, *ν* (Ni-O) 553, *ν* (Ni-N) 440; UV-Vis (DMSO, nm) (*ε*, cm^−1^ mol^−1^ dm^3^): 296 (10 900), 305 (11 900), 337 (19 200), 376 (15 000), 599 (50); ESI-HRMS: calcd. for C_26_H_16_N_4_O_2_Cl_2_Ni: [M + H]^+^ = 545.0077, found [M + H]^+^ = 545.0068.

#### 2.3.12. [2-(2-hydroxy-phenyl)-1H-benzimidazol-5-yl]-phenyl-methanone nickel (II) (**C_12_**)

Yellow powder, yield: 72%; M.p.: greater than 300°C; IR (KBr, cm^−1^): *ν* (-NH) 3050, *ν* (-C = N) 1618, *ν* (C = C) 1527, *δ* (-NH) 1479, *ν* (C-O) 1253, *ν* (Ni-O) 473, *ν* (Ni-N) 422; ESI-HRMS: calcd. for C_40_H_26_N_4_O_2_Ni: [M + Na]^+^ = 707.1205, found [M + Na]^+^ = 707.1296; UV-Vis (DMSO, nm) (*ε*, cm^−1^ mol^−1^ dm^3^): 379 (28 800), 594 (60).

#### 2.3.13. 2-(1H-benzimidazol-2-yl)-phenol silver (I) (**C_13_**)

Ligand **L_1_/L_5_** (1.2 mmol) was dissolved in distilled ethanol (10 ml) in a two-neck round-bottom flask. Then, a solution of AgNO_3_ (0.6 mmol) was added and the colour of the mixture changed immediately. The mixture was stirred at room temperature. After 3 days, a precipitate crystallized and was filtered from DMF. The product is the pink powder, yield: 78%; M.p.: greater than 300°C; IR (KBr, cm^−1^): *ν* (-NH) & *ν* (-OH) = 3336, *ν* (-C = N) 1605, *ν* (C = C) 1531, *δ* (-NH) 1469, *ν* (C-O) 1231, *ν* (Ag-N) 438; ESI-HRMS: calcd. for C_26_H_20_N_4_O_2_Ag: [M + H]^+^ = 527.0637, found [M + H]^+^ = 527.0710; UV-Vis (MeCN, nm) (*ε*, cm^−1^ mol^−1^ dm^3^): 281 (6200), 306 (8000), 320 (15 000); ^1^H-NMR (500 MHz, DMSO-*d_6_, δ*): 13.17 (s, 1H, -NH), 12.86 (s, 1H, -OH), 8.07–8.05 (d, 1H, Ar-H, *J* = 7.5 Hz), 7.72–7.63 (m, 2H, Ar-H), 7.40–7.37 (t, 1H, Ar-H, *J* = 7 Hz), 7.29 (s, 2H, Ar-H), 7.06–7.00 (m, 2H, Ar-H).

#### 2.3.14. 5-chloro-2-(2-trifluoromethyl)-phenyl-benzimidazol silver (I) (**C_14_**)

Grey powder, yield: 63%; M.p.: greater than 300°C*;* IR (KBr, cm^−1^): *ν* (-NH) 3064, *ν* (C = N) 1624, *ν* (C-N) 1317, *ν* (Ag-N) 472; ESI-HRMS: calcd. for C_28_H_16_F_6_Cl_2_Ag: [M + H]^+^ = 699.9707, found [M + H]^+^ = 699.9754. UV-Vis (DMSO, nm) (*ε*, cm^−1^ mol^−1^ dm^3^): 203 (55 800), 284 (24 400); ^1^H-NMR (500 MHz, DMSO-*d_6_, δ*): 13.46 (s, 1H, -NH), 7.94–7.92 (m, 1H, Ar-H), 7.82–7.74 (m, 4H, Ar-H), 7.69 (s, 1H, Ar-H), 7.36–7.34 (dd, 1H, *J* = 8.5 Hz, *J* = 1.5 Hz).

### 2.4. Computational details

Geometry optimizations of the ligands and complexes were carried out using Gaussian 09 [33] at the B3LYP/6-31 + G(d,p) level of theory [34,35]. The relativistic effective core potential, GEN (LANL2DZ), was used as a result of transition metal atoms [36,37]. Harmonic vibrational calculations were also performed under the identical function to confirm that the obtained structures had a global minimum on the potential energy surface. The solvent effects were considered using the continuum model of the solvation model based on density. The molecular information (geometries and frequencies) for all species is presented in electronic supplementary material, table S1.

The UV-Vis absorption spectra were simulated for the ligands in the Franck–Condon region (denoted as FC-UV). The lowest excited-state geometries were optimized using time-dependent density functional theory. The harmonic vibrational frequencies of both the ground and excited states are carefully verified, for which no imagination frequency is expected. A small benchmark of different density functionals with various exchange-correlation contributions, including global hybrid functionals (B3LYP, PBE0 and M05-2X) and range-separated hybrid functional (CAMB3LYP and wB97XD) were chosen to study the quality of the vibrationally resolved electronic spectra, as previously recommended [38]. The 6-311 + G(d,p) basis set was applied for all elements [39–41]. The solvent effect was studied using the polarizable continuum model [42,43], for the same solvent used in the experimental measurement, that is, DMSO and MeCN, depending on the molecules. The vibrationally resolved spectra within the harmonic approximation are then computed for one-photon electronic absorption spectra following the adiabatic Hessian approach [44]. For this theory, readers are invited to consult previous reports [45,46]. Finally, electronic spectra were obtained with half-width at half-maximum and resolution (HwHm/Res) of 400/50 or 200/20 cm^−1^.

### 2.5. Assay of anti-proliferation activities

Free ligands and metal-based complexes of benzimidazole derivatives were dissolved in 0.1% (v/v) DMSO to obtain various concentrations. Cisplatin was used as the standard drug, and DMSO was used as a blank control. All cells were grown in RPMI 1640 supplemented with 10% fetal bovine serum, 100 U ml^−1^ of penicillin and 100 µg ml^−1^ streptomycin in a 5% CO_2_ atmosphere for 48 h. After that, the cells were seeded in 96-well plates at a density of 10^4^ cells/well. After 24 h, the cells were treated with a culture medium containing the tested compounds in the concentration range. After 72 h, cell viability was determined as mitochondrial succinate dehydrogenase (SDH) activity using 0.5 mg ml^−1^ of the 3-(4,5-dimethylthiazol-2-yl)-2,5-diphenyltetrazolium bromide (MTT) test as a marker of viable cells and incubated at 37°C and 5% CO_2_ for 4 h, following the procedure described by Mosmann [47]. Acidified isopropanol was added to all wells and mixed thoroughly to dissolve the formazan crystals. The produced purple solution was spectrophotometrically measured at a wavelength of 570 nm using a Multikan™ microplate reader. The concentration of each compound was tested in triplicate. The proliferation inhibition (%PI) was calculated using the following formula:

$$\text{\%}\mathrm{PI}=100\text{–}[(A_{\mathrm{sample}}/A_{\mathrm{control}})\times 100].$$

Compounds with PI values greater than or equal to 50% were used to measure and calculate the IC_50_ values using nonlinear regression analysis.

### 2.6. Stability test of the complexes in PBS buffer

The complexes were tested for the solubility in the solvent such as water, ethanol, MeCN, DMF, DMSO and chloroform, qualitatively. Moreover, the stability of the complexes with IC_50_ values lower than 10 µM (**C_1_**, **C_3_** and **C_14_**) was examined by UV-Vis spectrometry using a UV-Vis Jasco V630 spectrophotometer. PBS buffer solution contains: NaCl (137 mM), KCl (2.7 mM), Na_2_HPO_4_ (10 mM) and KH_2_PO_4_ (2 mM), pH = 7.4. Samples were dissolved in PBS solution with 5% DMSO added to aid solubility, resulting in a final complex concentration of 20 µM. Spectra (between 200 nm and 750 nm) were recorded at room temperature in interval times (*t* = 0, 2, 4, 6, 8, 10, 12, 14, 16, 18, 20, 22 and 24 h) [48].

## 3. Results and discussion

### 3.1. Synthesis and characterization

**Scheme 1.**
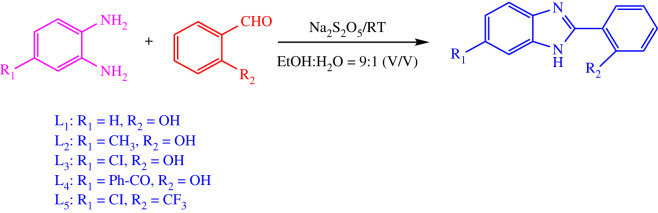
Synthesis reaction scheme of benzimidazole derivatives.

**Scheme 2.**
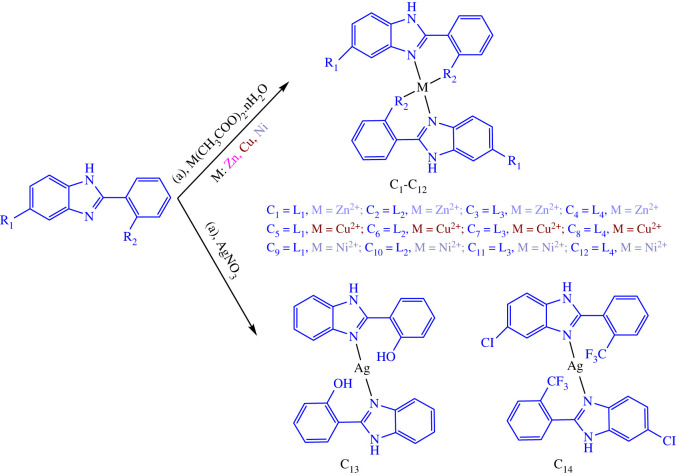
Synthesis reaction scheme of transition metal complexes of benzimidazole-derived moieties.

The benzimidazole derivatives shown in scheme 1 were synthesized by a condensation reaction between o-phenylenediamine and benzaldehyde in the presence of Na_2_S_2_O_5_ as an oxidation agent [31]. The yields obtained after purification ranged from 70% to 90%. Metal complexes of the benzimidazole-derived organic framework were synthesized from metallic salts, that is, M (II) (M = Cu, Zn, Ni), and Ag (I) in ethanol solution in a molar ratio of 2 : 1 (scheme 2). The obtained products were of various colours and morphologies, corresponding to their chemical nature, as indicated in the experimental section. In order to elucidate their structures and properties, the free ligands and complexes were characterized by spectroscopic and spectrometric analysis, such as FT-IR, UV-Vis, NMR, HR-ESI-MS and ICP-OES. However, it was impossible to obtain a suitable single crystal for XRD, despite various trials. We can only provide PXRD data. In addition, high-resolution mass spectrometry (HRMS) of the complexes revealed that molecular ion peaks in good agreement between the calculated and experimental *m/z* values contribute to supporting their structures.

### 3.2. Calculated structures of the compounds

**Figure 1.**
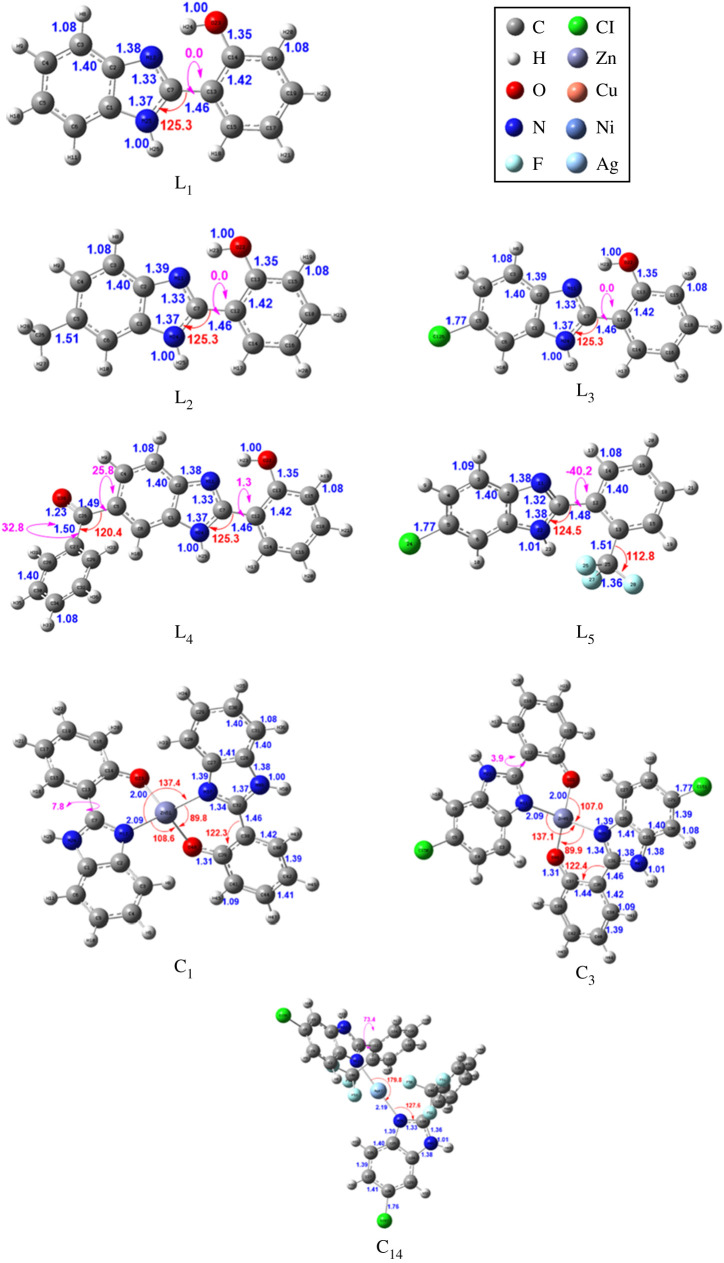
Optimized structures of ligands (**L_1_**–**L_5_**) and three selected complexes (**C_1_**, **C_3_** and **C_14_**), calculated at B3LYP/6-31 + g(d,p) level in DMSO solvent. The LANL2DZ basis set is used for the metal, and only important geometrical parameters (in angstroms and degrees) are provided.

Electronic calculations and experimental data provide valuable information to confidently confirm the structures of the ligands and also contribute to predicting the configuration of the complexes. In particular, figure 1 shows the optimized structures with key geometrical parameters (bond length in angstroms and angle in degrees) of the ligands (i.e. **L_1_**–**L_5_**) and selected complexes **C_1_**, **C_3_** and **C_14_**, which were calculated at the B3LYP level in DMSO solvent. For the other compounds, the data are provided in the electronic supplementary material. Detailed information, such as Cartesian coordinates and harmonic wavenumbers, is provided for all compounds in the electronic supplementary material. As shown in figure 1, ligands **L_1_** and **L_3_** are planar, while **L_2_**, **L_4_** and **L_5_** are non-planar because of the steric bulk of the substituents (i.e. -CH_3_, -C(=CO)Ph and -CF_3_ moieties, respectively). Metal (II) complexes **C_1_**–**C_12_** have four coordinates with two X-N and two X-O bonds, where X = Zn, Cu and Ni. In these complexes, the X-N bonds are found slightly longer than the X-O ones (e.g. approx. 2.09 versus 2.00, 2.01 versus 1.94 and 1.93 versus 1.88 Å for Zn, Cu and Ni complexes, respectively), and the angle ∡(NXO) is approximately 90^°^, where N and O are on the same ligand. On the other hand, the Ag (I) complexes **C_13_** and **C_14_** have the most prolonged Ag-N bonds (approx. 2.20 Å and have a linear structure).

### 3.3. FT-IR spectra

The FT-IR spectra of ligands **L_1_**–**L_5_** and complexes **C_1_**–**C_14_** are provided in table 1. The FT-IR spectra of ligands **L_1_**–**L_4_** show sharp bands at 3324, 3247, 3330 and 3273 cm^−1^, assigned to the intramolecular hydrogen bonding between the imine and nitrogen and oxygen atoms of the phenyl group [30]. The *ν*(C = N) imine group vibrations of benzimidazole rings appeared at 1631, 1639, 1633 and 1613 cm^−1^. However, in the **C_1_**–**C_12_** complexes, *ν*(C = N) vibrations shift down by 6–35 cm^−1^ compared with the corresponding ligands, indicating the coordination of the nitrogen atom to the metal. The absence of the sharp bands at 3330–3273 cm^−1^ and the appearance of the broad *ν*_NH_ bands at 3113–3041 cm^−1^ are attributed to complex formation [49]. All the characteristic *ν*_C = N_ peaks are shifted to lower frequencies by 11–37 cm^−1^, indicating the coordination of the nitrogen atom to the metal. In addition, the appearance of a weak band in the range of 553–422 cm^−1^ is attributed to the stretching vibrations *ν*_M-O_ and *ν*_M-N_ [50], respectively.

**Table 1.** FT-IR spectral data of the ligands and complexes. Vibration frequencies are in cm^−1.^

compound	*ν*_OH_ & *ν*_NH_	*ν*_C = N_	*ν*_C = C_	*δ*_N-H_	*ν*_C-N_	*ν*_C-O_	*ν*_M-O_	*ν*_M-N_
L_1_	3324	1631	1589	1491	1319	1261	–	–
L_2_	3237	1639	1540	1487	1321	1261	–	–
L_3_	3330	1633	1585	1489	1314	1257	–	–
L_4_	3273	1613	1573	1487	1294	1259	–	–
L_5_	3052 *ν*_NH_	1620	1583	1433	1314	–	–	–
C_1_	3108 *ν*_NH_	1625	1532	1477	1310	1252	539	427
C_2_	3054 *ν*_NH_	1604	1531	1477	1311	1249	517	428
C_3_	3058 *ν*_NH_	1603	1526	1477	1306	1249	508	422
C_4_	3087 *ν*_NH_	1622	1526	1477	1311	1247	503	435
C_5_	3057 *ν*_NH_	1602	1554	1478	1314	1259	474	438
C_6_	3049 *ν*_NH_	1604	1535	1479	1306	1261	469	438
C_7_	3113 *ν*_NH_	1602	1554	1476	1310	1255	523	439
C_8_	3052 *ν*_NH_	1617	1550	1475	1316	1254	476	441
C_9_	3098 *ν*_NH_	1605	1569	1481	1307	1262	468	441
C_10_	3056 *ν*_NH_	1605	1539	1481	1307	1262	469	441
C_11_	3041 *ν*_NH_	1605	1563	1479	1302	1253	553	440
C_12_	3050 *ν*_NH_	1618	1527	1479	1361	1253	473	422
C_13_	3336	1605	1531	1469	1285	1231	–	438
C_14_	3064 *ν*_NH_	1624	1546	1420	1317	–	–	472

### 3.4. Ultraviolet–visible spectra

The electronic absorption spectra of the free ligands and complexes were measured in DMSO or MeCN at room temperature, and the absorption bands are listed in table 2. Ligands **L_1_**–**L_5_** show specific absorption bands in the 206–297 nm and 319–337 nm regions, corresponding to the $\pi \to \pi^{*}$ transitions of aromatic rings and the $n\to \pi^{*}$ transitions of the C = N group in the benzimidazole ring, respectively. Such specific absorption bands of ligands were confirmed by the calculated data obtained from the Franck–Condon method. Unfortunately, the absorption of the complexes cannot be similarly calculated because of the very high computational cost of obtaining the excited states of transition metal complexes. For all complexes, a slight redshift in the old bands and the appearance of new peaks were observed. This is due to the combination of nitrogen–metal and oxygen–metal charge transfer. Furthermore, the d-d band of Cu(II) complexes in the 631–656 nm ranges is assigned to the ^2^T_2_→^2^E transition, supporting a tetrahedral distortion owing to the Jahn–Teller effect [50], and Ni(II) complexes exhibit two weak bands at 589 and 695 nm, caused by ^3^A_2_ (F) →^3^T_1_ (F) and ^3^A_2_ (F) → ^3^T_2_ (F) [51]. The UV-Vis spectra of Zn (II) and Ag (I) complexes changed slightly in the ultraviolet region compared with their ligands. Because Zn (II) and Ag (I) compounds have no unpaired d-electrons, no absorption band was found in the visible region.

**Table 2.** Electronic absorption spectral data of the compounds. The numbers in parentheses are calculated data using the FC-UV model with the M05-2X functional. The italicized numbers in parentheses are assigned the molar extinction coefficient values corresponding to each *λ*_max_.

compounds	electronic absorption bands' *λ*_max_ (nm)	charge transfer bands d-d
L_1_	293 (*9300*), 319 (13 300), 334 (12 100) (298, 310, 331)	–
L_2_	296 (*9800*), 322 (15 200), 337 (14 100) (300, 320, 337)	–
L_3_	297 (*9400*), 322 (16 300), 336 (15 000) (300, 319, 335)	–
L_4_	337 (16 200)	–
L_5_	206 (24 000), 292 (11 800) (330, 360)	–
C_1_	212 (33 500), 291 (*7100*), 317 (12 900), 330 (15 000), 363 (*1500*)	–
C_2_	241 (47 700), 290 (*7100*), 318 (*7000*), 334 (*7800*), 365 (*3200*)	–
C_3_	242 (42 000), 319 (*8100*), 335 (*7800*)	
C_4_	237 (42 800), 375 (28 800)	
C_5_	297 (17 100), 318 (12 100), 334 (13 500), 356 (21 800), 368 (21 100)	640
C_6_	300 (18 900), 323 (13 200), 358 (14 900), 371 (14 200)	641
C_7_	302 (15 100), 321 (10 900), 337 (19 200), 359 (24 600)	631
C_8_	380 (25 000)	656
C_9_	292 (16 100), 318 (15 300), 332 (15 600), 370 (15 600)	590, 687
C_10_	295 (12 400), 303 (12 800), 321 (12 500), 338 (16 900), 370 (17 800)	590, 676
C_11_	296 (10 900), 305 (11 900), 337 (19 200), 376 (15 000)	599
C_12_	379 (28 800)	594
C_13_	281 (*6200*), 306 (*8000*), 320 (15 000)	–
C_14_	203 (55 800), 284 (24 400)	–

### 3.5. Nuclear magnetic resonance spectra of the ligands

The ^1^H-NMR data of the ligands and complexes were measured in *d_6_*-DMSO at room temperature. In the reported ^1^H–NMR spectra of ligands **L_1_**–**L_4_**, two signals of OH and NH protons appeared in the region of 12.00–13.50 ppm (13.17 and 13.14 ppm for **L_1_**, 13.18 and 13.05 ppm for **L_2_** and 13.38 and 12.68 ppm for **L_4_**), while a broad singlet at 12.74 ppm was assigned to the NH proton for **L_3_** [52]. Ligand **L_5_** exhibited only a singlet for the NH proton at 12.99 ppm. The aromatic protons in ligands **L_1_**–**L_5_** shifted in the range of 6.99–8.10 ppm with different multiplet signals since various substitutions on the ring affected the phenolic ring. In particular, for **L_2_**, a singlet signal is observed at 3.23 ppm for the methyl group.

**Figure 2.**
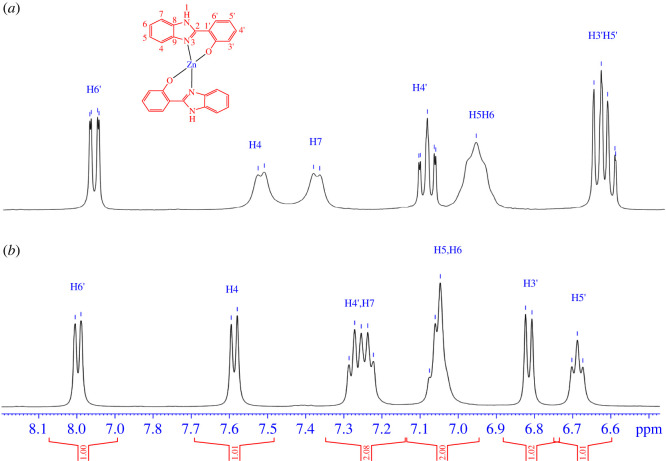
The ^1^H-NMR spectrum of ligand **L_1_** (*a*) and complex **C_1_** (*b*) in *d_6_*-DMSO.

To form the complexes, it is necessary to establish whether the coordination mode can occur via the N atom of the imidazole ring and the O atom of the phenolic group. This mode exerts effects on the chemical shifts of the aromatic protons. Such protons mostly appear in the high-field resonance signals in the region of 6.67–8.04 ppm due to the shifts of the electron density from metal to amine and the phenolic group. For example, the aromatic protons in the **C_1_** complex are shielded and absorbed at an upfield in the range of 6.69–8.00 ppm (compared with ligand **L_1_** at 7.00–8.06 ppm) in figure 2. It is noteworthy that the chemical shifts of the neighbouring protons of the mode are significantly influenced by H_3’_, H_5’_ and H_4_ positions. The H_3’_ and H_5’_ signals for ligand **L_1_** are given multiplet signals at 7.05–7.00 ppm, whereas the signals for complex **C_1_** are split into two distinct signals. In particular, the H_3’_ resonates at 6.82 ppm as a doublet, coupling to the proton H_4’_, while H_5’_ appears at 6.69 ppm as a triplet, coupling to the protons H_4’_ and H_6’_. Conversely, proton H_4_ moves from the upfield and the disappearance of the H atom of the phenolic group is found. The NH signal is ambiguous in DMSO solvents in any case. Ligand **L_1_**, acting as a bidentate ligand, is coordinated with the Zn (II) ion by the N (3) atom of the imidazole ring and the oxygen atom of the phenolic group. Similar changes were observed in the proton NMR spectrum of complex **C_3_**. Nevertheless, it is difficult to fully elucidate the influence of various substituents in the mode from adjacent protons, particularly the **C_2_** and **C_3_** complexes. Based on these results, the coordination mode of 2-(1*H*-benzimidazol-2-yl)-phenol to Zn (II) ion is expected to appear through the N (3) atom of the imidazole ring and the oxygen atom of the phenol group. Therefore, the zinc (II) ion has four coordinates, and the configurations of metal complexes are stereotypical of 2-(1*H*-benzimidazol-2-yl)-phenol ligand, which is observed for Cu (II) and Ni (II) complexes [53].

Comparing the ^1^H-NMR spectra of Ag (I) complexes with the corresponding ligands, minor shifts were observed for the aromatic ring protons. It should be noted that signals of the NH and OH protons of **C_13_** were distinct peaks at 13.17 and 12.86 ppm while ligand **L_1_** has overlapping signals at 13.16 ppm. Moreover, the singlet at 13.46 ppm is assigned to the signal of the NH protons of two benzimidazole rings in complex **C_14_** (compared with ligand **L_5_** at 12.99 ppm). Such signals support the linear geometry of the Ag coordination with the N atom of the pyridine ring. However, it is impossible to observe the proton signals of the Cu (II) and Ni (II) complexes in the ^1^H-NMR spectrum because of their paramagnetic characteristics.

### 3.6. Mass spectra

The molecular ion fragments in mass spectrometry were used to elucidate the probable formulae of the complexes. These results showed that the formation of the complexes occurs through a central metal that links two benzimidazole-derived molecules. For example, the mass spectral data for the copper (II) complexes **C_5_**–**C_7_** displayed ionic peaks at [M + H]^+^ = 482.0751, 511.1145 and 551.0226, respectively. The Cu (II) complexes **C_5_** and **C_6_** experience the disappearance of one molecule of **L_1_** and **L_2_**, respectively, and the appearance of a new fragment obtained [M-L_1_ + H_2_O] (C_13_H_11_N_2_O_2_Cu + H_2_O) at *m/z* 290.0152 for **C_5_** and [M-L_2_ + H_2_O] (C_14_H_13_N_2_O_2_Cu + H_2_O) at *m/z* 304.0325 for **C_6_**. Demetallation was recorded in these complexes, leading to the isolation of the metal-free ligand **L_1_** (*m/z* = 211.0918). Likewise, the Zn (II), Cu (II), Ni (II) and Ag (I) complexes **C_1_**–**C_3_**, **C_7_** and **C_9_**–**C_14_** displayed two dominant ionic fragments in the data, including a central metal (II) coordinated with a bidentate ligand and a peak of free ligands. On the other hand, compounds **C_4_**, **C_8_** and **C_12_** show pseudo-molecular ions, with peaks at [M + Na]^+^ 713.1153, 712.1142 and 707.1196, respectively. This indicated that the compounds were unstable and could open during ESI-MS analysis.

Finally, according to the detailed analyses, spectral data (FT-IR, ESI-MS, UV-Vis, ^1^H-NMR and ICP-OES) are in good agreement with the correlative structures of the complexes.

### 3.7. Powder X-ray diffraction studies

PXRD studies were carried out for all complexes (excluding **C_13_**). Indexing of the diffraction patterns was displayed using X'Pert HighScore Plus 3.0 software and the Miller indices (hkl) along with observed and calculated 2*θ* angles, relative intensities, and d values are shown in the electronic supplementary material. The type of crystal system, cell volume and lattice parameters of these compounds were calculated from the indexed data and are listed in table 3. Cu(II), Zn(II) and Ni(II) complexes were found to have orthorhombic structures, while the Ag(I) complex had a monoclinic structure. The crystal structures of similar types of these complexes have been published as orthorhombic and monoclinic ones [26,54–56]. In addition, the Debye–Scherrer equation *D* = *Kλ*/*β*Cos *θ*, where *D* is the particle size, *K* is the dimension shape factor, *λ* is the X-ray wavelength (0.15406 Å), *β* is the full-width at half-maximum of the diffraction peak and *θ* is the diffraction angle was used to calculate the average crystalline sizes of the complexes. The complexes have a crystalline size ranging from 25 to 58 nm, suggesting that they are in the nanocrystalline phase.

**Table 3.** The unit cells parameters from PXRD data of synthesized complexes.

Cps.	lattice parameters				volume (Å^3^)	crystallite size D (nm)	crystal system
	a (Å)	b (Å)	c (Å)	*β* (^◦^)			
C_1_	19.8970	11.9732	15.1742	90	3614.9612	39	orthorhombic
C_2_	18.5016	11.4730	26.1260	90	5545.7362	29	orthorhombic
C_3_	12.4798	19.5284	18.4492	90	4496.2642	58	orthorhombic
C_4_	12.5011	23.9274	28.2044	90	8436.4669	26	orthorhombic
C_5_	20.6901	12.3628	8.5790	90	2194.4015	29	orthorhombic
C_6_	10.8746	15.8411	17.8172	90	3069.2911	33	orthorhombic
C_7_	13.8515	23.0660	18.6709	90	5965.3283	32	orthorhombic
C_8_	18.6261	40.8152	10.3898	90	7898.6168	25	orthorhombic
C_9_	6.7427	12.7095	23.4648	90	2010.8476	40	orthorhombic
C_10_	14.6837	13.7124	23.6071	90	4753.2605	40	orthorhombic
C_11_	24.9036	23.9060	7.4031	90	4407.4020	30	orthorhombic
C_12_	6.4630	13.7838	25.1842	90	2243.5269	26	orthorhombic
C_14_	24.6221	8.5098	30.2032	93	6319.3127	37	monoclinic

### 3.8. *In vitro* cytotoxic activity

The *in vitro* cytotoxic activity of the ligands and metal complex-based benzimidazole scaffold were screened in three human cancer cell lines, A549 (human lung adenocarcinoma epithelial cell line), MDA-MB-231 (human breast adenocarcinoma cell line) and PC3 (human prostate carcinoma cell line) by using MTT (3-(4,5-dimethylthiazol-2-yl)-2,5-diphenyltetrazolium bromide) assay. The cytotoxicity efficacy of the compounds was expressed as IC_50_ values, as shown in table 4. Generally, the results of anti-proliferation activity show that the complexes have better anti-proliferative activity than the ligands (IC_50_ greater than 100 µM), with the IC_50_ values in the range of 5.82–80.83 µM, except ligand **L_5_**, which exhibits the highest anti-proliferation activity in the MDA-MB-231 and PC3 cell line assay, with the IC_50_ values 23.9 ± 0.9 and 23.4 ± 0.6 µM, respectively. Remarkably, the complexes **C_1_**, **C_3_** and **C_14_** possessed anti-cancer activity better than the reference drug cisplatin against A549 cells with their IC_50_ values of 8.9 ± 0.6, 8.7 ± 1.0, 5.8 ± 0.8 and 16.5 ± 1.0 µM, respectively, while the IC_50_ values of **C_1_**, **C_3_**, **C_4_**, **C_8_** and **C_14_** were lower than 10 µM and that of cisplatin (IC_50_ = 19.4 ± 1.7 µM) on MDA-MB-231 cell line.

**Table 4.** IC_50_ values of the ligands, complexes and cisplatin against three cancer cell lines.

Cps.	IC_50_ values (µM)		
	A549	MDA-MB-231	PC3
L_1_	>100	93.0 ± 1.34	>100
L_2_	>100	>100	>100
L_3_	>100	>100	>100
L_4_	>100	>100	>100
L_5_	>100	23.9 ± 0.9	23.4 ± 0.6
C_1_	8.9 ± 0.6	9.7 ± 0.8	10.4 ± 0.9
C_2_	43.9 ± 1.1	13.6 ± 0.8	27.1 ± 1.2
C_3_	8.7 ± 1.0	9.3 ± 0.7	9.8 ± 0.9
C_4_	63.3 ± 1.3	9.8 ± 0.8	55.6 ± 1.9
C_5_	>100	>100	>100
C_6_	18.3 ± 0.6	40.1 ± 1.1	15.6 ± 0.8
C_7_	>100	>100	>100
C_8_	34.8 ± 1.1	9.8 ± 0.2	18.3 ± 0.9
C_9_	69.6 ± 1.2	50.4 ± 1.7	52.8 ± 1.5
C_10_	80.8 ± 1.8	25.6 ± 1.0	>100
C_11_	>100	14.6 ± 0.8	82.1 ± 1.5
C_13_	15.00 ± 0.8	18.4 ± 0.7	20.7 ± 0.7
C_14_	5.8 ± 0.8	6.1 ± 1.0	7.0 ± 0.9
Cisplatin*	16.5 ± 1.0	19.4 ± 1.7	**

*Cisplatin as a reference drug.

It was found that Zn (II) complexes (**C_1_** and **C_3_**) are sensitive to all cancer cell lines, with IC_50_ values as low as 8.7–10.4 µM. This showed that the Cu (II) and Ni (II) complexes have less activity than the Zn (II) chelates. Cu (II) complexes (**C_5_** and **C_7_**) exhibited the lowest anti-proliferation activity among the complexes, with IC_50_ values greater than 100 µM. The resemblance of the anti-tumour activity of the Ni (II) chelates was lower than that of the Zn (II) complexes, with IC_50_ varies in the range of 14.6–82.1 µM. In particular, Ag (I) complexes, that is, complexes **C_13_** and **C_14_**, coordinated by ligands **L_1_** and **L_5_**, respectively, have significantly increased anti-cancer activity in all cancer cell lines. While the IC_50_ values of **C_13_** are in the range of 15.00–20.7 µM, the lowest IC_50_ values are obtained for **C_14_**, being 5.8 ± 0.8, 6.1 ± 1.0 and 7.0 ± 0.9 µM, for A549, MDA-MB-231 and PC3 cell lines, respectively. This illustrates that an appropriate choice of the benzimidazole-derived scaffold and the metal ions plays a crucial role in designing metallodrugs with selectivity and sensitivity against A549, MDA-MB-231 and PC3 cell lines. In addition, the structure–activity relationship was determined by comparing complex **C_14_** with **C_13_** and others, and the substituents of chlorine and trifluoromethyl groups in benzimidazole derivatives can boost the anti-proliferative activity against cell lines. It is noteworthy that three cancer cell lines, A549, MDA-MB-231 and PC3, were concomitantly inhibited by complexes **C_1_**, **C_3_** and **C_14_**, with IC_50_ < 10 µM. Therefore, these complexes can be considered multi-targeted anti-cancer agents.

The complexes were not insoluble in ethanol, water and chloroform and they dissolved completely in DMF and DMSO. In addition, MeCN is selectively soluble for the complexes **C_1_**, **C_2_**, **C_3_**, **C_4_** and **C_13_**. According to the results of *in vitro* cytotoxic activity above, the complexes **C_1_**, **C_3_** and **C_14_** were evaluated in PBS buffer (pH = 7.4) at a concentration of 20 µM between 200 and 750 nm for 24 h at room temperature to determine the stability in a physiological environment. This test demonstrated the possibility of transformation such as hydrolysis, reduction and precipitation of the complexes. According to electronic supplementary material, figure S85, no change was observed in the UV-Vis bands of complexes **C_1_** and **C_14_** (electronic supplementary material, figures S85*a*,*c*). Although there is a decrease in absorbance of UV-Vis spectrum of **C_3_** leading to the disappearance of the bands after 8 h, suggesting the decay of **C_3_**, it was stable over the first 8 h in this buffer without significant change in the spectrum (electronic supplementary material, figure S85*b*).

The cytotoxic activity of the complexes is proposed by the binding ability of benzimidazole moieties to DNA grooves via the interactions such as hydrogen bonds, electrostatic interactions and π–π stacking [57], one of the reasons assumed to cause apoptosis [58,59]. In addition, the effects of metal complexes on human cancer cell lines are previously reported to enhance the delivery of the active ligands to target intracellular sites[60], and the metal acts as a carrier and organic-ligand stabilizing agent until it approaches its targets. At the same time, organic carriers can protect the metal and avoid side-effects in its transit toward the second target of biological action [61].

Thus, the combined effects could result in the proliferation of bioactivities or activate new mechanisms of action [62]. On the other hand, the binding modes of the complexes toward DNA depend on the difference of metal ion Zn(II), Cu(II), Ni(II) and Ag(I). The transition metal cores show attachment to the major grooves since they exhibit binding affinity to nucleotide bases of DNA caused by the coordination bond between the transition metal centres and N-7 of guanine and/or adenine or N-3 of thymine and/or cytosine [7].

Besides, the results of anti-tumour activities of Zn (II) complexes have recorded plenty of evidence that displays an intriguing link between zinc and malignant cells. The growth of cancer cells can be inhibited by increasing zinc levels [63,64]. Typically, zinc is the second most abundant trace element in the human body [65]. Hence, the physiological importance of zinc reflects its presence in plenty of enzymes with over 300 biological functions and its regulation in over 2000 zinc-dependent transcription factors [66,67]. For instance, Ishii *et al*. [68] reported that zinc exhibits the catalytic regulation role of aminopeptidase N, whose dysregulation has been observed in numerous types of human cancer such as breast cancer, prostate cancer and non-small-cell lung cancer and is related to invasion in malignancies [69]. Moreover, previous results have shown that the tetrahedral geometry of the Zn (II) complex is inserted between the base pairs of DNA by one of the ligand molecules due to the Schiff base's planarity, whereas the other remains perpendicular, probably extra binding interaction with the DNA strands [70].

From the above results, the coordinate between the transition metal and benzimidazole moieties showed a significant increase in cytotoxicity, and complexes may be candidates for multi-targeted anti-cancer agents in the future. These compounds are the first examples of metal complexes of 2-(1*H*-benzimidazol-2-yl)-phenoxy-derived scaffolds with cytotoxic activity in human cancer cell lines A549, MDA-MB-231 and PC3.

## 4. Conclusion

Five free ligands and 14 transition metal complexes containing benzimidazole derivatives were designed, synthesized and characterized in this study. Multiple structural analyses revealed that metal M (II) (M = Zn, Cu, Ni) complexes adopt a distorted tetrahedral coordination geometry, while Ag (I) complexes have linear coordination. The detailed structures, together with the electronic absorption spectra of the ligands, were also calculated using electronic structure calculations to complement the experimental data. Moreover, *in vitro* evaluation showed that most of the complexes enhanced anti-cancer activity in comparison with the free ligands against three human cancer cell lines, A549 (human lung adenocarcinoma epithelial cell line), MDA-MB-231 (human breast adenocarcinoma cell line) and PC3 (human prostate carcinoma cell line). Specifically, complexes **C_1_**, **C_3_** and **C_14_** with the IC_50_ values of 5.8–10.4 µM showed the best anti-proliferative activity and better than the reference drug, cisplatin, whose IC_50_ values are 16.5 ± 1.0 and 19.4 ± 1.7 µM on A549 and MDA-MB-231 cell lines, respectively. Thus, they could be the new promising agents in chemotherapeutic applications and will be investigated on the other cancer cell lines and the particular mechanism of action in further research.

## Ethics

This article does not contain any studies involving animals performed by any of the authors.

## Data Availability

The datasets supporting this article have been uploaded as part of the electronic supplementary material [71].
